# Clinical Profile and Outcomes of Multisystem Inflammatory Syndrome in Children: A Multicentric Observational Study

**DOI:** 10.7759/cureus.28821

**Published:** 2022-09-06

**Authors:** Bishwajit Mishra, Bibhudatta Mishra, Arjit Mohapatra, Vidya Patwari, Shobha D Malini, Mamta Panda, Suryakanta Swain

**Affiliations:** 1 Pediatrics, Hitech Medical College, Bhubaneswar, IND; 2 Pediatric Critical Care, Jagannath Hospital, Bhubaneswar, IND; 3 Pediatrics and Neonatology, Asian Institute of Public Health University, Bhubaneswar, IND; 4 Pediatrics, Jagannath Hospital, Bhubaneswar, IND; 5 Community Medicine, Maharaja Krushna Chandra Gajapati Medical College, Berhampur, IND

**Keywords:** echocardiography, covid-19.aneurysm, shock, coronary artery aneurysm, hyperinflammation, myocarditis, mis-c

## Abstract

Background and objective

Multisystem inflammatory syndrome in children (MIS-C) is a postinfectious, generalized, hyperimmune state and is potentially lethal. There is scarce data on the clinical presentation and epidemiology of MIS-C in India. In light of this, we conducted this study to describe clinical presentations and outcomes in children diagnosed with MIS-C.

Methodology

This was a 15-month hospital-based prospective observational study conducted in the Departments of Pediatrics at Jagannath Hospital and Hitech Medical College, Bhubaneswar. The study included all patients diagnosed with MIS-C and treated at these hospitals between May 1, 2020, and August 31, 2021. The inclusion criteria were as follows: patients who were reverse transcription-polymerase chain reaction (RT-PCR)-positive, antibody-positive, or had known contact with those infected with coronavirus disease 2019 (COVID-19). We reviewed patient medical records to collect demographic data such as age, sex, body mass index (BMI), duration of illness, clinical symptomatology, findings of initial echocardiography, and outcomes. We followed each case for three months. We analyzed descriptive statistics using percentages and means and conducted the statistical analysis using SPSS Statistics for Windows, Version 25.0. (IBM Corp., Armonk, NY).

Results

A total of 30 cases were included in the study, consisting of 16 boys (53.3%) and 14 girls (46.7%). The mean age of the study population was 6.7 years, and 43% had a BMI in the overweight range. All patients (100%) had a fever, 66.7% had lethargy (n=20), and 64.3% (n=19) had abdominal symptoms in the form of vomiting, diarrhea, and abdominal pain. Respiratory distress at admission was found in 16 cases (53.3%), while hypotension at admission was found in 18 (60%) cases. Our population's average duration of pediatric ICU stay was 3.7 ± 1.2 days, and the average duration of inotropy was 2.2 ± 0.5 days. Fifteen cases (50%) required only oxygen support; 10 (33%) required noninvasive ventilation, and only one patient required invasive ventilation. Twenty-two patients (74%) needed fluid boluses. Outcomes of coronary artery dilatations were favorable, regressing to normal (Z-score <2.5) in affected patients within 90 days of follow-up.

Conclusions

MIS-C has myriad presenting signs, symptoms, and severity. It is often associated with circulatory failure or shock. However, most patients demonstrated good early outcomes, improved left ventricle (LV) function, normalization of coronary abnormalities, and no mortality. This study provides additional data on the clinical presentation of MIS-C and highlights the importance of close, long-term follow-up monitoring of this patient population.

## Introduction

Multisystem inflammatory syndrome in children (MIS-C) has emerged as a serious consequence of severe acute respiratory syndrome coronavirus 2 (SARS‑CoV‑2) infection in the pediatric population [1]. A case series from the United Kingdom, Italy, and France [2,3] has reported that MIS primarily affects children aged one month to 18 years, and most patients had no underlying comorbid conditions. Symptoms such as cough or troubled breathing are not prominent features in MIS-C patients, unlike those with coronavirus disease 2019 (COVID-19). MIS-C is a postinfectious, generalized hyperimmune state that is potentially lethal. MIS-C shares some clinical features with Kawasaki disease (KD), and MIS-C management protocols often influence KD protocols [4]. Data on the clinical presentation and epidemiologic characteristics of children with MIS-C remain limited but are the focus of ongoing research. The objective of this study was to describe the presentations and outcomes in children diagnosed with MIS-C.

## Materials and methods

Study design and data collection

This was an observational study conducted over 15 months in the Departments of Pediatrics at Jagannath Hospital and Hitech Medical College, Bhubaneswar, involving patients treated from May 1, 2020, to August 31, 2021. Demographic data such as age and sex and clinical information, including duration of illness, clinical symptomatology, findings of initial echocardiography, and outcomes up to 90 days following admission, were collected. The study was approved by the Institutional Ethical Committee at Jagannath Hospital and Hitech Medical College (approval no: HMCH/IEC/2022/158).

Inclusion criteria

All children aged one month to 18 years who fulfilled the World Health Organization criteria for MIS-C were included in the study and followed up for three months after the initial presentation. The criteria for MIS-C consisted of fever for at least three days and any two of the following aspects: (i) skin rashes or bilateral nonpurulent conjunctivitis or mucocutaneous inflammation signs, (ii) hypotension or shock, (iii) features of myocardial dysfunction, pericarditis, valvulitis, or coronary abnormalities (including echocardiography findings or elevated troponin/N-terminal pro-B-type natriuretic peptide), (iv) coagulopathy (by prothrombin time, partial thromboplastin time, and elevated d-dimers), and (v) acute gastrointestinal (GI) problems (e.g., diarrhea, vomiting, or abdominal pain). The criteria also included high inflammatory markers such as erythrocyte sedimentation rate (ESR), C-reactive protein (CRP), or procalcitonin in the absence of other apparent microbial causes of inflammation including bacterial sepsis, staphylococcal or streptococcal shock syndromes [5]. The epidemiological link to SARS-CoV-2 in these patients was confirmed by reverse transcriptase-polymerase chain reaction (RT-PCR) testing (with or without a positive antibody test) or antibody positivity (with negative or unknown RT-PCR results).

Exclusion criteria

Patients without features of MIS-C or those older than 18 years of age were excluded. Forty-five patients were eligible for inclusion, but 15 were lost to follow-up. The remaining 30 patients were included in the study.

Statistical analysis

Data were gathered using Microsoft Excel spreadsheets (Microsoft Inc., Redmond, WA). Descriptive statistics, such as percentages for categorical variables and means for continuous variables, were used. Collected data were collated, and appropriate statistical analysis was performed using SPSS Statistics for Windows, Version 25.0. (IBM Corp., Armonk, NY).

## Results

Our study population consisted of 30 patients: 16 (53.3%) boys and 14 (46.7%) girls. The mean age of the patients was 6.7 years. Thirteen patients (45%) were overweight. Fever was found in all 30 cases (100%), lethargy in 20 cases (66.7%), and abdominal symptoms (e.g., vomiting, diarrhea, and abdominal pain) were found in 19 (63.3%) cases (Table 1). Bilateral conjunctival congestion was present in 25 cases (83.3%). Eighteen patients (60%) had oral mucosal changes, eight patients (26.7%) had unilateral cervical lymphadenopathy, and 23 patients (76%) had neutrophilic leucocytosis.

**Table 1:** Symptomatology of MIS-C patients (n=30) MIS-C: multisystem inflammatory syndrome in children; ENT: ear, nose, and throat

Constitutional	Symptoms	Number of patients with symptoms	Percentage of patients with symptoms
	Fever	30	100%
	Lethargy	20	66.7%
	Lymphadenopathy	8	26.7%
	Limb edema	8	26.7%
Gastrointestinal	Vomiting/diarrhea/pain in the abdomen	19	63.3%
Eye	Bilateral conjunctival congestion	25	83.3%
	Bilateral conjunctivitis	24	80%
ENT	Neck pain	4	13.3%
	Oral mucosal changes	18	60%
	Neck swelling	6	20%
Respiratory	Cough	7	23.3%
	Shortness of breath	26	86.7%
Neurological	Convulsions	1	3.3%
	Mental changes	25	83.3%
Dermatological	Rash	20	66.7%
	Extremity edema	14	46.7%

The mean platelet count in the study population was 120,000 platelets/µL; the mean CRP was 61.2 mg/L, and procalcitonin was 2.5 ng/mL (Table 2). Twenty-two cases (73.3%) had transaminitis. The mean value of ferritin was 254.57 ng/mL (7-140 ng/mL), the mean lactate dehydrogenase level was 607.0 U/L (<245 U/L), and the mean D-dimer level was 4885.6 ng/mL (<500 ng/mL).

**Table 2:** Laboratory values (n=30) WBC: white blood cells; LDH: lactate dehydrogenase; ESR: erythrocyte sedimentation rate

Laboratory parameter		Average at admission	Average at discharge
Hematology			
	Hemoglobin (g/dl)	11.1	12.6
	WBC (/mm^3^)	13303.67	6715.488
	Neutrophil (%)	77.5	60.5
	Lymphocytes (%)	19.5	30.6
	Platelet (/mm^3^)	120,000	290,000
Liver and renal function			
	Albumin (g/L)	3.043	3.7
	Creatinine (mg/dL)	0.7993	0.39836
	LDH (U/L)	606.975	159.8627
Inflammatory markers			
	C-reactive protein (mg/L)	62	25.36
	Ferritin (ng/mL)	254.57	148.119
	Procalcitonin (ng/mL)	2.567	1.04369
Coagulation			
	D-dimer (ng/mL)	4885.567	2243.3387
	ESR (mm/h)	84.345	14.7413

Our population's average length of stay in the pediatric ICU (PICU) was 3.7 ± 1.2 days, and the average duration of inotropy was 2.2 ± 0.5 days (Table 3). Fifteen cases (50%) required only oxygen support; 10 cases (33%) required noninvasive ventilation [e.g., continuous positive airway pressure (CPAP) or bilevel positive airway pressure (BiPAP)], and two patients required invasive ventilation.

**Table 3:** Clinical profile of MIS-C patients (n=30) MIS-C: multisystem inflammatory syndrome in children; ICU: intensive care unit; CPAP: continuous positive airway pressure; BiPAP: bilevel positive airway pressure; IM: intramuscular; IVIG: intravenous immunoglobulin; LMWH: low-molecular-weight heparin

Treatment		Number of patients requiring treatment	Percentage of patients requiring treatment and duration
ICU duration		30	3.7 ± 1.2 days
Oxygen support		15	50.00%
CPAP/BiPAP		10	33.33%
Invasive ventilation		2	6.70%
Bolus fluid		22	73.30%
Vasopressor		18	60%
	Only adrenaline	15	50%
	Adrenaline with milrinone	3	10%
Duration of vasopressor support		18	2.2 ± 0.5 days
IM methylprednisolone		30	100%
IVIG	1 g/kg	5	16.70%
	2 g/kg	11	36.70%
Aspirin		30	100%
LMWH		2	6.70%
Outcome	Good	30	100%

Four cases (13%) had elevated amylase and lipase levels suggestive of acute pancreatitis, and one had necrotizing pancreatitis. Eighteen cases (60%) were treated with intravenous immunoglobulin (IVIG). All patients received two-dimensional echocardiography, and low ejection fraction was noted in 22 cases (73%) and pericardial effusion in 26 cases (87%). Ten cases (33%) had coronary dilatation, and none had an aneurysm. IVIG was administered to 16 patients (54%) at a dose of 1-2 g/kg in a single infusion over 12 to 48 hours. All 30 patients received methylprednisolone at doses ranging from 2 to 30 mg/kg/day according to disease severity. Given the cost of IVIG, we instituted this only for those patients who presented in shock with severe left ventricle (LV) dysfunction and coronary abnormalities. Vasopressor support was needed for a mean of 2.2 ± 0.5 days in 18 cases (60%), and an IV fluid bolus was needed in 22 cases (73.3%). All patients were given aspirin as soon as they could tolerate it orally. All patients had sinus tachycardia; none had sinus bradycardia or any major heart block. Depressed ejection fraction (45%) and pericardial effusion (87%) were common cardiac presentations of MIS-C. Dilatations of the coronary arteries (Z score >2.5) were found in 13 patients (33%), and none had a major aneurysm (Z score >5). All patients were discharged home, and no death was recorded. Outcomes of coronary artery dilatations were favorable, regressing to normal (Z-score <2.5) in affected patients within 90 days of follow-up.

## Discussion

We conducted this study to describe the presentations and outcomes of children diagnosed with MIS-C. We observed no meaningful differences in terms of gender, and our participants' demographic profile regarding age and the male-to-female ratio was similar to the findings of Whittaker et al., although their population’s median age (nine years) was older than that of our study population [6]. In most cases, GI symptoms like abdominal pain, vomiting, and diarrhea were present. The abdominal pain was so severe that patients were initially presumed to have appendicitis, which was later found to be MIS-C in several cases. Belhadjer et al. and Dasgupta et al. have reported urgent abdominal surgery in similar circumstances, and those cases were later determined to be mesenteric lymphadenitis [7,8]. Four of our cases had elevated levels of pancreatic enzymes, one of whom was diagnosed with necrotizing pancreatitis. The hyperinflammatory state seen in MIS-C may play a role in the pathogenesis of intestinal involvement. There is a similar known association between KD and GI manifestations, including appendicitis [9]. There may be a contributory role of the angiotensin-converting enzyme 2 receptors in its pathogenesis as they are expressed in the intestine, allowing SARS-CoV-2 to invade GI cells and cause inflammation [10].

Most of our patients had neutrophilic leucocytosis with low platelet count, lymphocytopenia, and elevated D-dimer levels. The average level of neutrophils was 77%, similar to the findings reported in a study on 99 children from New York (82.3%) [11]. Our population's mean total leukocyte count was 13,800/µL, and lymphocyte count was 19%, which is similar to the findings of Lagunas-Range et al.'s meta-analysis that found an association between the high white cell count, low lymphocyte count, low platelet count, elevated CRP, and disease severity [12]. The inflammatory markers like CRP, ESR, and procalcitonin were elevated in our study population. In adult studies, raised levels of CRP are associated with severity and mortality in COVID-19 patients [13]. Our mean D-dimer value was very high, similar to the elevated D-dimer levels in pediatric and adult patients with COVID-19 [14].

We used IVIG, anticoagulants, and steroids as the primary therapies for MIS-C. Previous studies suggest that this approach reduces the risk of coronary abnormalities in high-risk children [15-17]. We did not use remdesivir per Wang et al.'s warning against using remdesivir in patients who are PCR-negative for COVID-19 [18]. We used low-molecular-weight heparin at a prophylactic dose in sick PICU patients with severe headaches and very poor LV function.

Our study's cardiac presentations were similar to those reported in an Indian study but differed from those reported in a French study, where the course was more severe and had a greater need for close monitoring in patients where the initial echocardiography was unremarkable [19,20]. The reported mortality in another Indian series ranged from 0% to 27.5% [21].

Our study had several significant limitations. Our sample size was relatively small, and we did not perform a subgroup data analysis. Our center is a tertiary care referral hospital; therefore, findings in our population may not be representative of the overall spectrum of MIS-C in the general population.

## Conclusions

MIS-C, which may follow a symptomatic or asymptomatic COVID-19 infection, is a hyperinflammatory syndrome with a broad spectrum of clinical manifestations and variable severity. Thus, it is often challenging to recognize this heterogeneous disease in daily clinical practice. In our study, children diagnosed with MIS-C demonstrated good early outcomes with improved LV function and normalization of coronary abnormalities and without any mortality. Rapid diagnosis, multidisciplinary management, bedside echocardiography, and early suppression of systemic inflammation are associated with a favorable outcome.
